# NEMO Binds Ubiquitinated TANK-Binding Kinase 1 (TBK1) to Regulate Innate Immune Responses to RNA Viruses

**DOI:** 10.1371/journal.pone.0043756

**Published:** 2012-09-18

**Authors:** Lingyan Wang, Shitao Li, Martin E. Dorf

**Affiliations:** Department of Microbiology & Immunobiology, Harvard Medical School, Boston, Massachusetts, United States of America; Institut Pasteur, France

## Abstract

RIG-I-like receptors (RLR) are intracellular sensors utilized by nearly all cell types for recognition of viral RNA, initiation of antiviral defense, and induction of type I interferons (IFN). TBK1 is a critical kinase implicated in RLR-dependent IFN transcription. Posttranslational modification of TBK1 by K63-linked ubiquitin is required for RLR driven signaling. However, the TBK1 ubiquitin acceptor sites and the function of ubiquitinated TBK1 in the signaling cascade are unknown. We now show that TBK1 is ubiquitinated on residues K69, K154, and K372 in response to infection with RNA virus. The K69 and K154 residues are critical for innate antiviral responses and IFN production. Ubiquitinated TBK1 recruits the downstream adaptor NEMO through ubiquitin binding domains. The assembly of the NEMO/TBK1 complex on the mitochondrial protein MAVS leads to activation of TBK1 kinase activity and phosphorylation of the transcription factor, interferon response factor 3. The combined results refine current views of RLR signaling, define the role of TBK1 polyubiquitination, and detail the mechanisms involved in signalosome assembly.

## Introduction

An important aspect of host resistance against viral infections is the production of type I interferons (IFN). Cytosolic receptors, such as the RIG-I like receptors (RLR), sense viral RNA in nearly all cell types. Following RNA recognition, RLRs translocate onto a scaffold molecule termed MAVS which serves as a platform for coordinating downstream innate immune signaling [1], [2]. RLR engagement of MAVS leads to activation of downstream kinases and transcription factors, including TBK1 and interferon regulatory factor 3 (IRF3), respectively. Following RLR-MAVS interaction, TBK1, a constitutively and ubiquitously expressed serine-threonine kinase, catalyzes phosphorylation of IRF3 [3], [4], [5], [6]. However, the mechanisms by which RLR signals recruit and activate TBK1 are not well understood.

The importance of TBK1 to antiviral immunity is underscored by observations that several viruses evolved strategies to target or hijack this enzyme. For example, inhibition of TBK1 interactions with IRF3 by Borna disease virus P protein dampens the innate immune response [7], the Gn protein of pathogenic hantaviruses disrupts formation of TBK1 complexes, thereby blocking downstream responses required for IFN transcription [8], the γ_1_34.5 protein of herpes simplex virus inhibits TBK1 [9], and the hepatitis C virus NS3/4A protein interacts directly with TBK1 [10] to inhibit IFN production. Elucidating the biochemical mechanisms controlling assembly of TBK1 with other signaling intermediates can advance our understanding of the innate immune defense system and may reveal new targets of microbial pathogenesis.

Recently, TBK1 K63-linked polyubiquitination (pUb) was shown to be important for the LPS and RLR induced IFN production [11], [12], [13]. The E3 ligases Mind Bomb 1 and 2 (MIB1 and MIB2) couple K63-linked ubiquitin to TBK1 in response to RNA virus infection [13] while Ndrp1 ubiquitinates TBK1 in response to LPS [12]. However, the sites of ubiquitination and the molecular contribution of K63-linked polyubiquitin to RLR signaling remain unknown. We now analyze the TBK1 ubiquitination sites and demonstrate a molecular mechanism underlying the critical role of TBK1 pUb for recruitment of NEMO in early antiviral responses.

## Materials and Methods

### Cells and reagents

Murine embryonic fibroblasts (MEF) derived from *mib1* ^f/f^ mice [14] were donated by Dr. Y-Y. Kong (Seoul National Univ.). MIB1^f/f^ MEF were immortalized by transfection with SV40 LT. MEF derived from mice genetically deficient for TBK1 (Drs. Delhase and Nakanishi, Nagoya University), NEMO (Dr. Schmidt-Supprian, Harvard Medical School), and *Tnfr1* ^−/−^; *tbk1* ^−/−^ macrophages (Dr. Fitzgerald, Univ. Massachusetts) were generously provided.

Poly(I∶C)-LMW/LyoVec was purchased from Invivogen (San Diego, CA). Antibodies directed to IRF3, NEMO, phospho^396^S-IRF3, and ubiquitin were bought from Cell Signaling (Beverly, MA). Antibodies against TBK1 were purchased from Imgenex (San Diego, CA) and Millipore (Billerica, MA). Antibodies specific for FLAG, MYC and HA were purchased from Sigma Chemical Co (St. Louis, MO). Recombinant GST-IRF3 was purchased from Abnova (Taiwan). Antibody against phospho^172^S-TBK1 was obtained from BD Bioscience (San Jose, CA).

### Real-time PCR

mRNA was quantified using SYBR Green based real-time PCR. Total RNA was prepared using TRIzol Reagent (Invitrogen). Two µg RNA were transcribed into cDNA using QuantiTect reverse transcription kit (Qiagen). For one real-time reaction a 20 µl SYBR Green PCR reaction mix (Roche Applied Science), including 1/40 of the synthesized cDNA plus oligonucleotide primer pairs detailed elsewhere [15] were run on the LightCycler II (Roche). Controls were done in parallel without adding reverse transcriptase to rule out genomic DNA contamination. The comparative Ct method was used to determine relative mRNA expression of genes as normalized by the β-glucuronidase housekeeping gene.

### siRNA

The efficiency of the MIB2 siRNAs was validated elsewhere [13].

### Immunoblotting, immunoprecipitation

Western blot was performed as previously described [16]. All immunoprecipitations were performed using Pierce Direct IP kit. For ubiquitin immunoprecipitation, cells were lysed in RIPA buffer (Sigma) with N-ethylmaleimide (Calbiochem), protease inhibitor cocktail (Roche), and phosphatase inhibitor (Pierce). The cell lysates were incubated with the indicated antibody conjugated beads and mixed end-over-end at 4°C overnight. The beads were then 3× washed (5 min/wash) with the same buffer used for cell lysis. For affinity purification of protein complexes, stably transfected HEK293 cells were lysed in RIPA buffer (Sigma) plus 10 mM NaF, 1 mM Na_3_VO_4_, 0.5 mM DTT, and a cocktail of protease and phosphatase inhibitors (Pierce Biotechnology).

### *In vitro* kinase assays

For kinase assays FLAG-TBK1 and mutants were purified from HEK293 cells stably transfected with the respective FLAG-tagged constructs. FLAG-TBK1 or mutants (10 ng), GST-IRF3 (25 ng), 0.2 mM ATP were incubated in 1× Kinase Buffer (Cell Signaling) at 30°C for 60 min.

### Luciferase reporter assay, cell transfection, and infection

HEK293 cell transfections were performed using Polyfect (Qiagen) or Lipofectamine 2000 (Invitrogen) according to the manufacturer's protocol. MEFs and macrophages were transfected using Amaxa nucleofection according to the manufacturer's protocol (Lonza GmbH, Germany). The ISRE reporter (Stratagene) and luciferase assays were performed as recommended by the manufacturer (Promega, Madison, WI). Luciferase assays were performed using the Dual Luciferase reporter system (Promega) as detailed elsewhere [17]. Relative luciferase units (RLU) were measured and normalized against *Renilla* luciferase activity 48 hr after transfection. Values are expressed as mean ± SD of three experiments.

For cell infection 5 or 50 HA Sendai virus or the indicated multiples of infection (MOI) of vesicular stomatitis virus (VSV) were added. 1 µg/ml poly(I∶C)-LMW was transfected using LyoVec (Invivogen).

VSV-eGFP and VSV-Luc were kindly provided by S. Whelan (Harvard University). Sendai virus was purchased from Charles River (Cambridge, MA). Wild type adenovirus and Adeno-Cre were purchased from University of Iowa adenoviral core.

### Mass spectrometry

Samples were analyzed at the Beth Israel Deaconess Medical Center (Boston) mass spectrometry core facility.

## Results

### Virus-dependent TBK1 K63-linked ubiquitination sites

**Figure 1:**
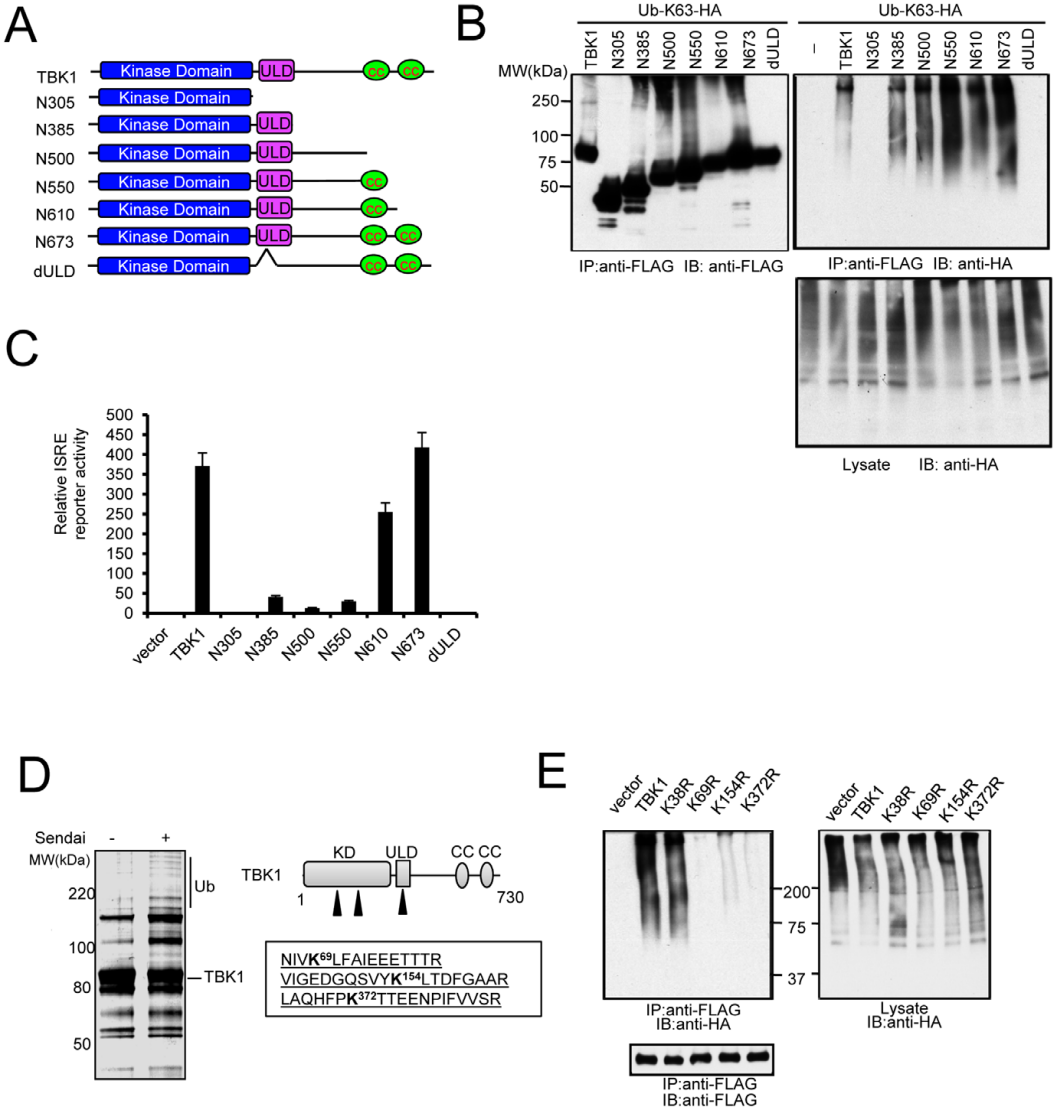
Mass spectrometry identifies TBK1 ubiquitination sites in response to RNA virus. (**A**) Map of various TBK1 truncation mutants showing the schematic structure of kinase domain, ubiquitin-like domain (ULD), and coiled coil domain (CC). (**B**) Kinase and ubiquitin-like domains are required for TBK1 activity. FLAG-tagged TBK1 truncation mutants were transfected with Ub-K63-HA into HEK293 cells. After 48 hr cells were collected, immunoprecipitated, and immunoblotted with the specified antibodies. Molecular weights are indicated. (**C**) ISRE reporter activity of HEK293 cells transfected with indicated TBK1 truncation mutants. (**D**) TBK1 complexes from control or Sendai infected 293 cells stably transfected with FLAG-TBK1 were affinity purified, then separated on a Nu-PAGE gel which was stained with Commassie blue. The fraction labeled Ub was sent for mass spectrometric analysis. Right panel shows the schematic structure of TBK1 kinase domain (KD), ubiquitin-like domain (ULD), and coiled coil domain (CC). Ubiquitination sites identified by mass spectrometry are indicated by arrowheads and peptides are detailed in the box. (**E**) HEK293 cells were cotransfected with designated FLAG-tagged TBK1 mutants and HA-tagged K63-only ubiquitin. Lysates were immunoprecipitated and immunoblotted as designated.

Various TBK1 truncation mutants were prepared to identify the domain required for TBK1 ubiquitination (Fig. 1A). TBK1 mutants were transfected into HEK293 cells with K63-only ubiquitin (containing a single lysine reside). The combination of the kinase domain and ubiquitin-like domain (ULD) were sufficient for TBK1 ubiquitination as the N385 deletion mutant lacking the C-terminal coiled coil domains was heavily ubiquitinated (Fig. 1B). Overexpression of TBK1(N385) induced low but significant levels of luciferase reporter activity driven by the interferon-stimulated response element (ISRE) which requires activation by IRF3 (Fig. 1C). ULD deletion (dULD) abolished TBK1 activity [18] and prevented TBK1 pUb (Fig. 1B, 1C).

To further identify the TBK1 ubiquitin acceptor site, we selected HEK293 cells stably expressing FLAG-TBK1. Two hr after infection with Sendai virus, *in vivo* ubiquitinated forms of FLAG tagged TBK1 were affinity purified and analyzed by multi-dimensional liquid chromatography coupled with tandem mass spectrometry. This analysis identified ubiquitin moieties on TBK1 residues K69, K154, and K372 (Fig. 1D). To examine the biologic properties associated with these sites, HEK293 cells were transfected with TBK1 K→R mutants along with K63-only ubiquitin. The K69R, K154R, and K372R mutants displayed reduced K63-linked pUb (Fig. 1E). The data suggest K69, K154, and K372 are acceptor sites for K63-linked ubiquitin although additional ubiquitin acceptor sites may exist. In contrast, the kinase inactive K38R mutant [3] was heavily ubiquitinated indicating that TBK1 catalytic activity is not required for K63-linked ubiquitination. The data also suggest ubiquitination precedes TBK1 transautophosphorylation [19].

### Ubiquitination sites are critical for TBK1 activity

**Figure 2:**
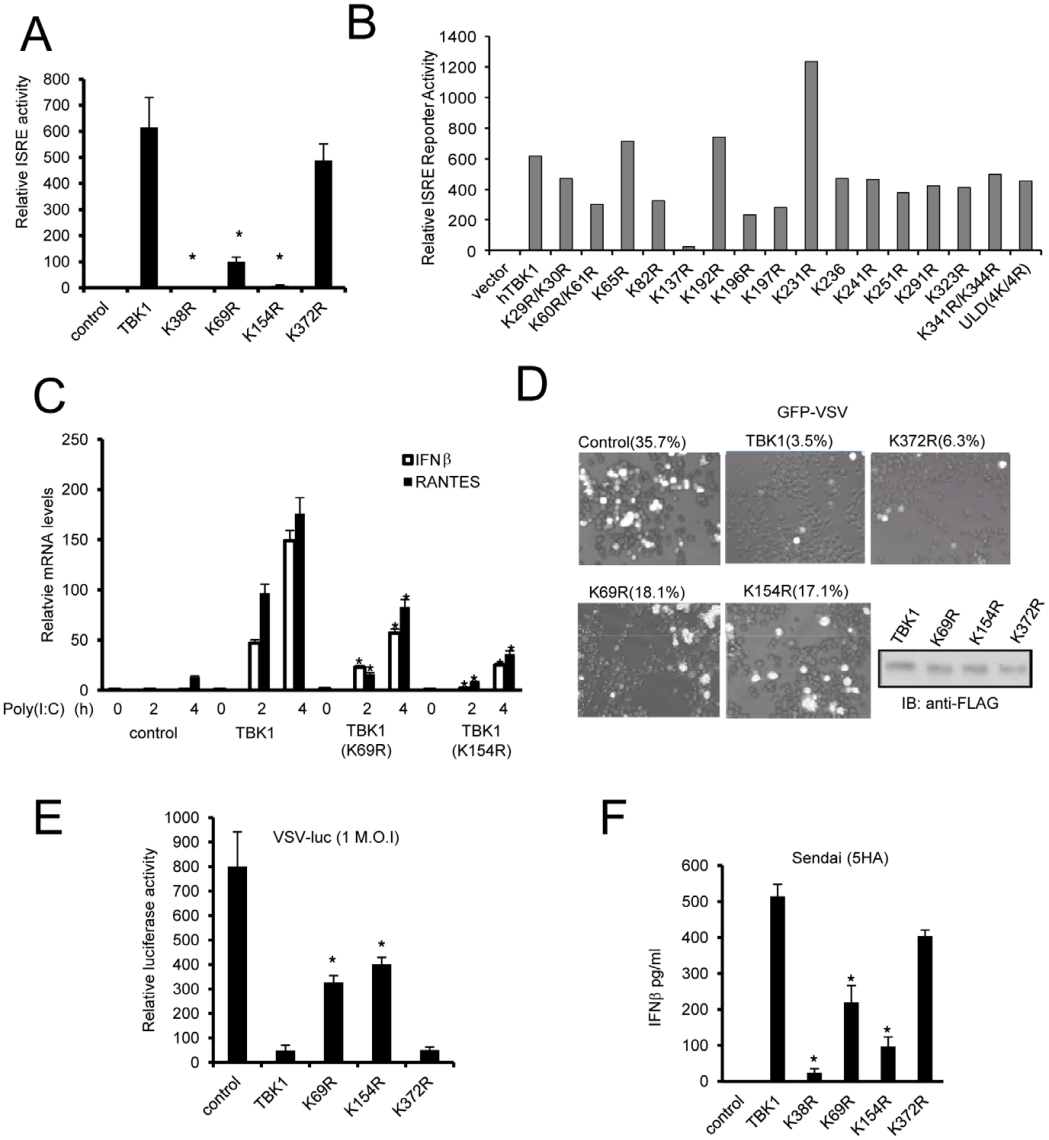
Mutation of ubiquitination sites impairs innate antiviral response. (**A**) ISRE reporter activity in control, FLAG tagged TBK1, and K→R mutant transfected HEK293 cells. Data present the mean ± SD of triplicate samples. Asterisk indicates P<0.01. (**B**) ISRE reporter activity of TBK1 K→R mutants. Various TBK1 mutants were transfected into HEK293 cells with ISRE and Renilla reporter plasmids. (**C**) *Tbk1^−/−^* MEFs were transfected with TBK1 and TBK1 mutants. After 48 hr cells were treated with 1 µg/ml poly(I:C) for the designated times. Relative levels of IFNβ and RANTES RNA are presented. Asterisk indicates P<0.01. (**D**)*Tnfr1* ^−/−^, *tbk1* ^−/−^ macrophages were transfected with FLAG-tagged TBK1 and TBK1 mutants. After 24 hr cells were infected with 0.1 MOI (multiple of infection) VSV-eGFP for 12 hr. Control TBK1 levels are included in the lower panel. (**E**) VSV-Luciferase (VSV-Luc) replication in HEK293 cells transfected with TBK1 or TBK1 mutants. Cells were infected with VSV-Luc at 1 MOI. Luciferase reporter activity was measured 18 hr after infection. (**F**) Induction of IFNβ in *tbk1* ^−/−^ MEFs transfected with indicated TBK1 constructs. Two days later cells were treated with 5 HA Sendai virus for 12 hr. Data represent the mean ± SD of triplicate samples. *P<0.05 was calculated by comparison to wild type TBK1.

We next examined the K69R, K154R, and K372R mutants to evaluate the impact of ubiquitination on TBK1 activity. The K69R and K154R mutations severely impaired TBK1-induced ISRE reporter activity (Fig. 2A). As expected the K38R construct which is known to lack kinase activity was completely unable to induce ISRE activity. In contrast, the K372R mutant showed a modest but insignificant reduction of reporter activity (Fig. 2A). To evaluate the potential roles of other lysine residues on ISRE reporter activity we mutated 18 additional conserved lysine residues. In addition, we examined a ULD(4K/4R) mutant in which all four lysines within the ULD were altered. Only K137R markedly reduced TBK1 activity (Fig. 2B). Alignment of the TBK1 kinase domain in the NCBI database of conserved domains predicts K137 is a conserved site for ATP binding and kinase activation. To evaluate the impact of K69 and K154 mutation on additional measures of TBK1 activity, *tbk1* ^−/−^ MEFs were transfected with wild type or mutant TBK1 and stimulated with poly(I∶C) (a synthetic RNA duplex). TBK1 mutation impaired stimulation of IFNβ and chemokine (RANTES) transcription (Fig. 2C). We also transfected these mutant constructs into TBK1 deficient macrophages and infected the cells with VSV-eGFP. The K69R and K154R containing cells display intermediate levels of protection compared with wild type or K372R TBK1 (Fig. 2D). Next we transfected mutant TBK1 constructs into HEK293 cells and then infected the cells with VSV containing a luciferase reporter (VSV-Luc). Wild type TBK1 and the K372R mutant inhibited viral replication while the K69R and K154R single mutants demonstrated weakened protection from viral infection compared to wild type TBK1 (Fig. 2E). In addition, *tbk1^−/−^* MEFs reconstituted with either K69R or K154R TBK1 produce less IFNβ following infection with Sendai virus than cells reconstituted with wild type or K372R TBK1 (Fig. 2F). Thus, mutation of the K69 or K154 TBK1 ubiquitin acceptor sites impairs antiviral activity and IFN production in response to infection.

**Figure 3:**
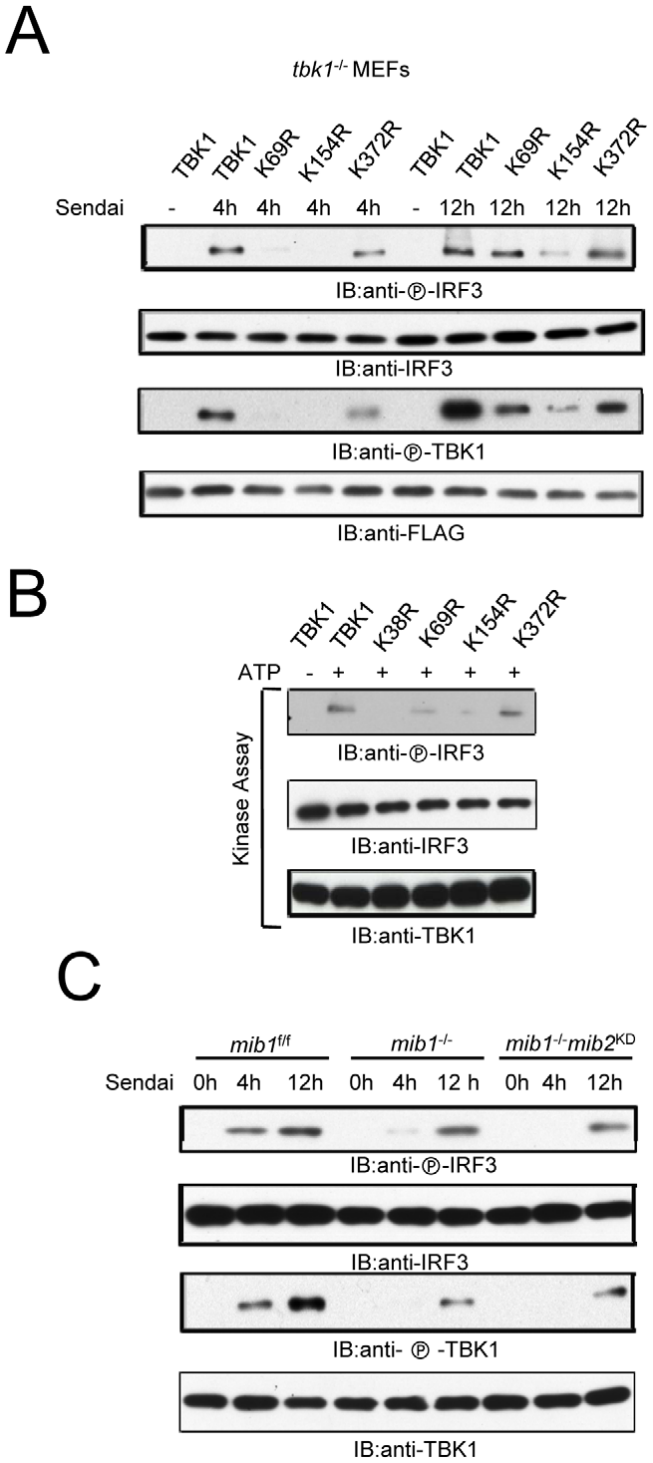
Ubiquitination regulates TBK1 kinase activity. (**A**) IRF3 and TBK1 phosphorylation in *tbk1* ^−/−^ MEFs transfected with indicated TBK1 constructs. Two days later cells were infected with Sendai virus. (**B**) *In vitro* kinase assay of TBK1 and TBK1 mutants using IRF3 as substrate. HEK293 cells were transfected with indicated FLAG-tagged TBK1 constructs after 48 hr TBK1 was purified with anti-FLAG. Purified TBK1 was examined for kinase activity using IRF3 as substrate. (**C**) *Mib1^f/f^*, *mib1* ^−/−^, or *mib1* ^−/−^ cells transfected with MIB2 siRNA (*mib2* ^KD^) were treated with 50 HA Sendai virus for the designated times. Cell lysates were immunoblotted as indicated.

To explore whether TBK1 mutations affect substrate phosphorylation, reconstituted *tbk1* ^−/−^ MEFs were infected with Sendai virus. Following viral infection K69R and K154R reconstituted cells displayed reduced enzyme activity as monitored by either TBK1 or IRF3 phosphorylation (Fig. 3A). The effects of K69 or K154 mutation were most apparent in the early (4 hr) response to viral infection. *In vitro* kinase assays using IRF3 as substrate support the conclusion that the K69R and K154R mutants display reduced catalytic activity (Fig. 3B). The combined data suggest these two ubiquitin acceptor sites are also critical for TBK1 kinase activity.

Because K69 and K154 sites reside in the kinase domain, inactivation of TBK1 kinase activity by mutation can alternatively be explained by disruption of kinase structure. To avoid disruption of the TBK1 kinase domain by mutation we used MIB1 and MIB2 double deficient cells to examine the role of ubiquitination on TBK1 activity. MIB1 is the primary E3 ligase for RLR-dependent K63-linked TBK1 pUb while the closely related MIB2 contributes less because of its low level of expression in MEFs [13]. As shown in Fig. 3C, Sendai virus-induced IRF3 and TBK1 phosphorylation were dramatically reduced in MIB1/MIB2 double deficient MEFs compared to control *mib1* ^f/f^ MEFs in early infection. MEFs deficient in MIB1 (*mib1* ^−/−^) alone displayed a reduction in IRF3 and TBK1 phosphorylation (Fig. 3C). Most TBK1 activity was restored 12 hr after viral infection in *mib1* ^−/−^ and MIB1/MIB2 double deficient MEFs (Fig. 3C). Taken together, the data suggest that TBK1 ubiquitination is essential for optimal early antiviral signaling and disruption of TBK1 ubiquitination impairs TBK1 activity.

### TBK1 recruits NEMO through K63-linked ubiquitin chains

**Figure 4:**
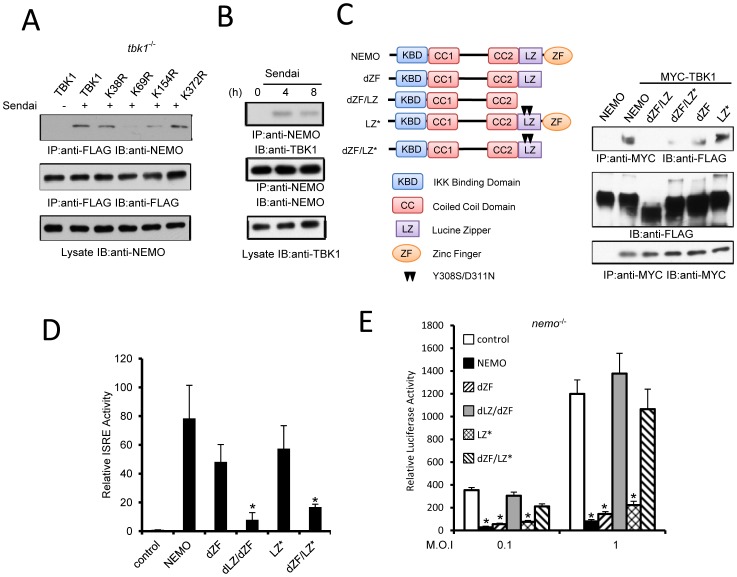
TBK1 K63-linked pUb recruits NEMO. (**A**) *tbk1* ^−/−^ MEFs transfected with indicated TBK1 constructs were infected with Sendai (2 hr) then cell lysates were immunoprecipitated with anti-FLAG antibody and immunoblotted as indicated. (**B**) MEFs infected with 5 HA Sendai virus were harvested at the indicated times and immunoprecipitated with anti-NEMO antibody. (**C**) Indicated FLAG tagged NEMO constructs were transfected with Myc-TBK1 into HEK293 cells. Cell lysates were immunoprecipitated with anti-Myc antibody and immunoblotted with indicated antibodies. NEMO constructs with zinc finger deletion (dZF), leucine zipper and zinc finger deletion (dZF/LZ), and Y308S and D311N point mutations (LZ*) are indicated. (**D**) HEK293 cells were transfected with the indicated constructs and ISRE reporter activity was assayed. Data represent mean ± SD of triplicate samples. Asterisk indicates P<0.01. (**E**) *Nemo* ^−/−^ MEFs were transfected with the indicated constructs after 24 hr cells were infected with VSV-Luc at indicated MOI. Luciferase reporter activity was measured 18 hr after infection. Data represent mean ± SD of triplicate samples. Asterisk indicates P<0.01.

NEMO can associate with TBK1 [13], [20], [21] and is critical for virus-induced IRF3 activation [13], [22], [23]. Therefore we examined whether the association between TBK1 and NEMO is dependent on K63-linked ubiquitination. After infection with Sendai virus the K69R and K154R point mutants show severely reduced NEMO binding (Fig. 4A). These results are supported by the finding that Sendai infection induces association between endogenous TBK1 and endogenous NEMO (Fig. 4B). NEMO has two ubiquitin binding domains (UBD) and both are required for IFN activation [22], [23] however, the targets of these UBD are unknown. To examine which UBD domain is required for association with TBK1 we transfected TBK1 with various NEMO mutants (Fig. 4C). Y308S/D311N mutations in the leucine zipper domain (LZ*) or zinc finger deletion (dZF) alone had little effect on TBK1 binding. However, the combination of Y308S/D311N mutation and zinc finger deletion (dZF/LZ*) or deletion of both UBD (dZF/LZ) prevented association between NEMO and TBK1 (Fig. 4C), suggesting both the LZ and ZF domains contribute to NEMO-TBK1 interaction. To examine the biological effects of UBD mutation, NEMO constructs were transfected into HEK293 cells and *nemo* ^−/−^ MEFs. The cells were then monitored for ISRE reporter activity (Fig. 4D) and VSV replication (Fig. 4E). Deletion of the ZF domain or mutation of the LZ had little impact on reporter activity or viral growth, while destruction of both UBD domains significantly reduced biological activity. The combined results suggest TBK1 associates with NEMO through both UBD.

**Figure 5:**
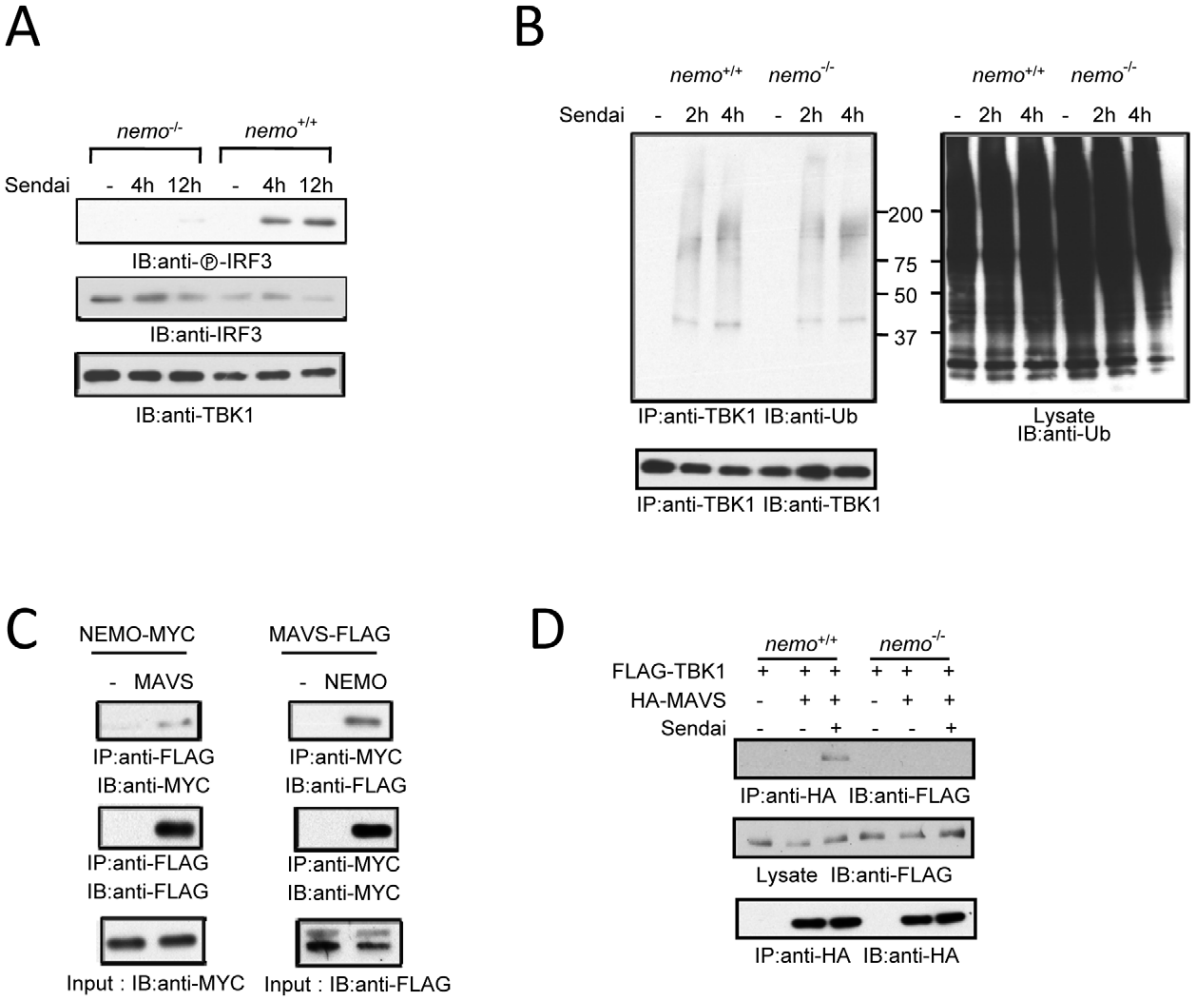
NEMO bridges TBK1 to MAVS. (**A**) IRF3 phosphorylation in Sendai infected wild type and *nemo* ^−/−^ MEFs. (**B**) TBK1 ubiquitination in Sendai infected wild type and *nemo* ^−/−^ MEFs. (**C**) Reciprocal immunoprecipitation between MAVS and NEMO. NEMO- MYC and MAVS- FLAG were transfected into HEK293 cells after 48 hr cells were collected. Cell lysates were immunoprecipitated and immunoblotted with the indicated antibodies. (**D**) Wild type and *nemo* ^−/−^ MEFs were cotransfected with FLAG-TBK1 and HA-MAVS after 48 hr cells were infected with 5HA Sendai virus. Cell lysates were immunoprecipitated and immunoblotted with the designated antibodies.

Next we examined TBK1 activation and ubiquitination in *nemo^−/−^* cells. As previously reported [22], [24], NEMO deficiency abolished virus-induced IRF3 phosphorylation (Fig. 5A). TBK1 is ubiquitinated following viral infection even in the NEMO deficient cells (Fig. 5B). The combined data indicate that NEMO acts downstream of TBK1 and suggests a mechanism by which the binding property of NEMO promotes signalosome assembly.

Using proteomic analysis we established that recognition of RNA induces association between the NEMO and MAVS proteins [13]. To confirm the requirement of NEMO for assembly of the MAVS complex, NEMO and MAVS were coexpressed in HEK293 cells. As shown in Fig. 5C, NEMO associates with MAVS by reciprocal immunoprecipitation. Furthermore, after Sendai virus infection TBK1 can coimmunoprecipitate with MAVS in wild type but not in NEMO deficient MEFs (Fig. 5D). Taken together, the data suggest that NEMO bridges TBK1 to MAVS through TBK1 K63-linked polyubiquitin chains.

## Discussion

TBK1 is regarded as a converging point for signaling from a diverse spectrum of microbial sensors [25], [26]. TBK1 recruitment to divergent upstream scaffolds such as MAVS, TRIF or STING appears to involve multiple mechanisms. Thus, distinct E3 ligases participate in RLR and LPS-mediated TBK1 activation [12], [13], while cytosolic DNAs activate TBK1 without apparent TBK1 ubiquitination [13], [27]. Defining the requirements for TBK1 recruitment to these different scaffolds is needed to better understand the molecular basis for TBK1 activation in different contexts. This report is the first to define ubiquitin acceptor sites critical for TBK1 function.

Ubiquitin-dependent mechanisms play a central role in transmitting signals required for activation of innate antiviral responses [28]. K63-linked ubiquitin provides various targeted proteins with an ability to engage in new protein interactions with molecules containing ubiquitin binding domains, thereby contributing to the formation of a functional signalosome. In the RLR signaling pathways, several proteins including TANK, RIG-I, MAVS, and TRAF3 undergo ligand-induced K63-linked ubiquitination. It appears that multicomponent complexes of polyubiquitinated intermediates are progressively assembled. The combinatorial addition of successive elements of the signalosome is a highly regulated process. Several distinct E3 ligases are reported to regulate ubiquitination following recognition of viral nucleic acids [29], [30], [31], [32]. In addition, the deubiquitinases CYLD and DUBA have been identified as negative regulators of IRF3 activation through deubiquitination of TBK1, RIG-I, or TRAF3 [11], [33], [34]. Thus, polyubiquitination emerges as a common theme in the RLR signaling pathway.

NEMO is critical for IFN production in response to RNA viruses [22], [23]. Previous models proposed that NEMO links TBK1 to the MAVS complex through an adaptor termed TANK [22], [23]. Subsequently, TANK deficient cells were shown to display normal responses to infection with RNA viruses [35]. Thus, TANK is not essential for MAVS signaling, however, we cannot exclude the possibility that TANK or redundant adaptors help stabilize MAVS-NEMO-TBK1 complexes. Here we provide evidence that viral infection induces endogenous association of TBK1 with NEMO and this interaction depends upon association of NEMO ubiquitin binding domains with K63-ubiquitinated TBK1. Although NEMO appears to be responsible for bringing TBK1 to the MAVS complex, how NEMO and other downstream members of the signalosome are recruited to the mitochondrial or peroxisome surface remains unresolved [36]. Recent evidence suggests that MAVS and IRF3 associated proteins, HSP90, GSK3β or IFIT3, may help stabilize TBK1 complexes [37], [38], [39] although the mechanisms are yet to be elucidated.

**Figure 6:**
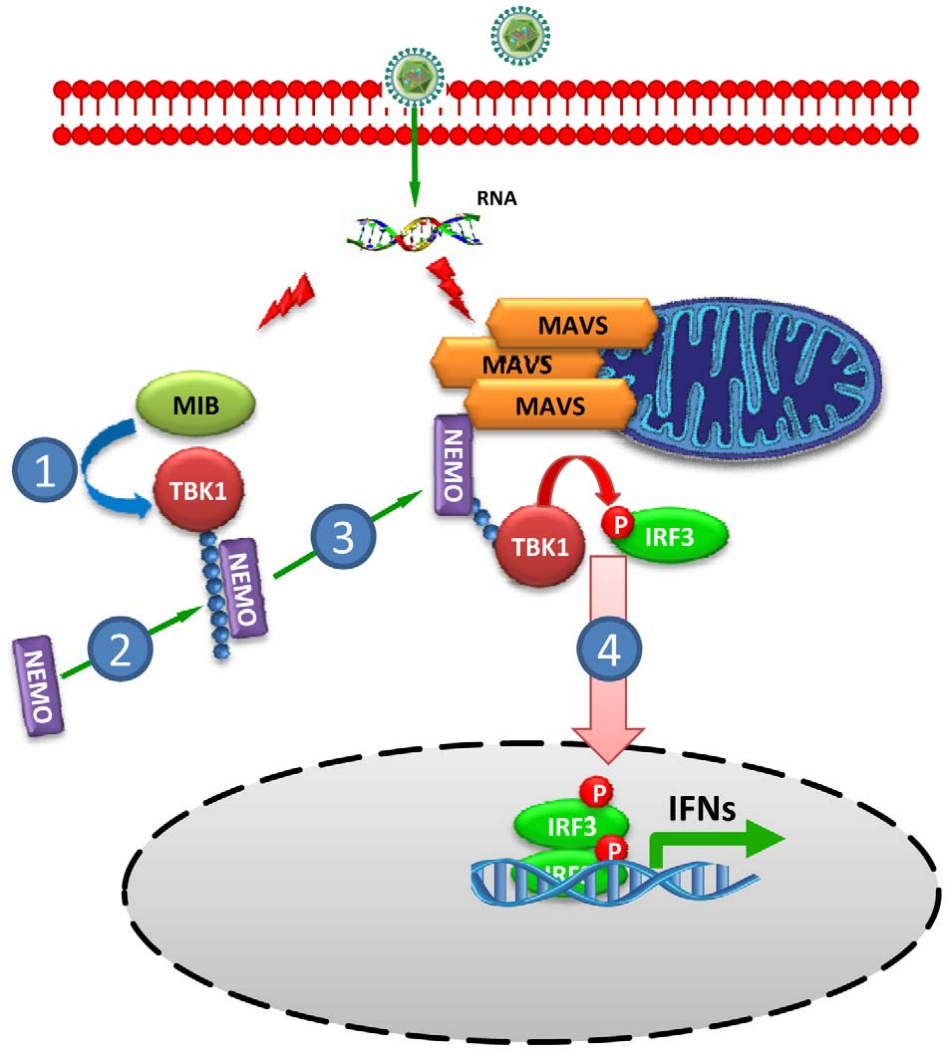
Model of TBK1 recruitment to MAVS. Double-stranded RNA and RNA viruses induce MIB-TBK1 association resulting in K63-linked TBK1 pUb. NEMO binds ubiquitinated TBK1 and the complex is recruited to MAVS. The MAVS signalosome serves as platform for TBK1 activation leading to IRF3 phosphorylation.

Taken together, we propose a new model for the molecular mechanism regulating TBK1 activation (Fig. 6). Recognition of viral RNA promotes association of MIB1 and/or MIB2 with TBK1 [13], resulting in MIB dependent K63-linked TBK1 ubiquitination. NEMO binds polyubiquitinated TBK1 and the NEMO-TBK1 complex is recruited to MAVS. Viral infection induces large-prion like MAVS aggregates implicated in IRF3 activation [40]. Virus-activated MAVS aggregates on the outer mitochondrial membrane, where it can serve as a platform for signalosome assembly linking TBK1 with its IRF substrate leading to activation of IRF3, and triggering transcription of IFN and other antiviral genes.

In summary, we identified sites of TBK1 ubiquitination in response to infection with RNA viruses and proposed a mechanism for assembly of a RLR-induced molecular complex responsible for IRF3 signal propagation.
